# Cold Atmospheric Plasma Improves the Therapeutic Success of Photodynamic Therapy on UV-B-Induced Squamous Cell Carcinoma in Hairless Mice

**DOI:** 10.3390/ph18060907

**Published:** 2025-06-17

**Authors:** Stephanie Arndt, Petra Unger, Irina Ivanova, Wolfgang Bäumler, Konstantin Drexler, Mark Berneburg, Sigrid Karrer

**Affiliations:** Department of Dermatology, University Medical Center Regensburg, 93053 Regensburg, Germany; petra.unger@ukr.de (P.U.); irina.ivanova@ukr.de (I.I.); wolfgang.baeumler@ukr.de (W.B.); konstantin.drexler@ukr.de (K.D.); mark.berneburg@ukr.de (M.B.); sigrid.karrer@ukr.de (S.K.)

**Keywords:** photodynamic therapy (PDT), 5-aminolaevulinic acid (ALA), cold atmospheric plasma (CAP), actinic keratosis (AK), squamous cell carcinoma (SCC)

## Abstract

**Background/Objectives:** Actinic keratosis (AK) occurs on sun-damaged skin and is considered a precursor to squamous cell carcinoma (SCC). Photodynamic therapy (PDT), using 5-aminolevulinic acid (ALA) and red light, is a common treatment for AK. However, its clinical efficacy for invasive tumors such as SCC is limited by the poor penetration and distribution of the photosensitizer. Cold atmospheric plasma (CAP), a partially ionized gas, increases skin permeability and exhibits anti-cancer properties through the generation of reactive oxygen species (ROS). In a previous study, CAP showed promising synergistic effects when combined with ALA-PDT for the treatment of SCC cells in vitro. The present study investigated the effects of combining CAP with ALA-PDT on cutaneous AK and SCC induced by ultraviolet B (UV-B) irradiation in SKH1 hairless mice. **Methods:** We compared various application sequences (CAP-ALA–red light, ALA–red light–CAP, and ALA-CAP–red light) against conventional ALA-PDT using visual, histological, and molecular assessments of the affected skin. **Results:** The results demonstrated that combined treatments strongly inhibited the growth of UV-B-induced skin lesions. TUNEL staining revealed increased apoptosis following both single and combined therapies, while Ki-67 staining indicated reduced keratinocyte proliferation and diminished DNA damage in treated areas. mRNA expression analysis showed the upregulation of apoptosis-related genes (*p16^INK4a^*, *p21^CIP1^*) alongside enhanced anti-tumor immune responses (*IL-6*, *IL-8*) in the affected tissue samples. Notably, the combined treatment enhances the therapeutic effect, whereas the sequence of application does not seem to be relevant for therapeutic efficacy in vivo. **Conclusions:** Overall, these results suggest that CAP may enhance the anti-tumor effect of conventional ALA-PDT, supporting previous findings on SCC cells.

## 1. Introduction

Actinic keratosis (AK) is a common precancerous skin condition characterized by rough, scaly patches. These patches develop as a result of prolonged exposure to ultraviolet (UV) radiation from the sun or artificial sources [1]. Estimates suggest that the annual incidence in the general population is about 10 to 25 per 1000 people. However, it can be significantly higher in high-risk groups (such as older adults or individuals with fair skin) [2,3]. The lifetime prevalence of AK is estimated to be around 20 to 40%, with even higher rates seen among older adults and those with significant sun exposure [4]. AK can progress to squamous cell carcinoma (SCC), a type of skin cancer that is invasive. SCC can metastasize and cause serious health issues if left untreated [5].

The treatment of AK is crucial to prevent potential progression to SCC. To date, conventional photodynamic therapy (PDT) is considered one of the most effective treatment options for multiple AK lesions associated with field cancerization [6]. PDT uses visible light of certain wavelengths to selectively destroy tumor tissue while preserving the surrounding healthy structures. The therapy begins with the application of a precursor involved in the natural production of heme, such as 5-aminolevulinic acid (ALA). This compound is applied to the skin tumor for several hours [7]. During this period, the precursor penetrates the skin and is preferentially converted into protoporphyrin IX (PpIX) within the tumor cells. PpIX serves as a potent photosensitizer [8]. When exposed to red light, PpIX generates reactive oxygen species (ROS), which are highly reactive molecules that can damage cellular components [7]. A critical aspect of the effectiveness of PDT is its ability to induce oxidative stress, particularly through mitochondrial damage. This damage triggers the release of cytochrome c from the mitochondria into the cytoplasm, initiating a cascade that leads to apoptosis. One of the major advantages of PDT is its selective targeting of cancer cells, allowing for the effective treatment of tumors while minimizing damage to adjacent healthy tissue [9]. This selectivity makes PDT a valuable option for treating superficial tumors and certain types of non-melanoma skin cancer. However, topical PDT is not currently approved for treating cutaneous SCC due to concerns about its potential invasiveness and risk of metastasis [10]. Despite this, numerous reports indicate that topical PDT can produce favorable outcomes in the treatment of superficial and microinvasive SCC, especially for patients or lesion sites that cannot tolerate surgery [11,12,13,14,15]. While the effectiveness of PDT is limited by factors such as depth of light and photosensitizer penetration and distribution, it is important to find alternative methods to optimize treatment conditions.

Cold atmospheric plasma (CAP) treatment involves the application of ionized gas at room temperature, which generates reactive species such as ROS and nitrogen species (RNS). These reactive species can interact with biological tissues, leading to various effects including antimicrobial activity, the modulation of cellular responses, and the potential enhancement of therapeutic outcomes.

CAP has received considerable attention in cancer research in recent years. The anti-cancer effect of CAP on AK and SCC has been reported in various preclinical studies and clinical case studies [16,17,18]. The selective growth-reducing effect of CAP treatment on SCC cells was observed in vitro [19,20] and in vivo [16,21], and CAP was able to halt the progression of UV-B-induced SCC-like skin lesions without affecting healthy skin [16,22]. However, the biological mechanism of the anti-tumor potential of CAP has not yet been sufficiently elucidated, which is why no major clinical studies have yet been conducted in tumor patients.

In addition to its anti-tumorigenic property, CAP has the ability to temporarily destabilize the skin barrier [23]. Thus, the transdermal delivery of substances can be facilitated by a mechanism similar to electroporation [24,25,26,27,28,29]. The permeability-promoting property together with the anti-tumorigenic effect of CAP may improve the efficacy of conventional therapies such as PDT.

The synergistic effects of those combined treatments were already observed in an in vitro study with cervical cancer cells [30]. The authors were able to demonstrate superior PDT activity on CaSki cells due to improved targeting. They also described the more efficacious inhibition of cervical cancer cell growth due to increased intracellular generation of ROS supported by CAP treatment [30]. Recently, we were able to show in various oral and cutaneous SCC cell lines that combined treatment with CAP and PDT can significantly improve therapeutic success [31]. Overall, these results suggest that combined CAP-PDT treatment is an efficacious new therapeutic modality that may be considered for the treatment of AK, SCC, and other tumor diseases.

The aim of this study was to show whether the synergistic therapeutic effects of CAP and PDT, which have so far only been shown in vitro, can also be confirmed in vivo, with a view to their subsequent clinical use in the treatment of AK and also possibly SCC.

## 2. Results

We used a well-established model of UV-B-induced SCC skin lesions to compare the efficacy of various treatment approaches. These included conventional ALA-PDT once a week for 4 weeks (group 2A), CAP treatment twice a week for 4 weeks (group 3A), and several combined treatment sequences: CAP followed by ALA–red light (group 4A), ALA–red light followed by CAP (group 5A), and ALA-CAP–red light (group 6A). The treatments were compared to untreated tumor-bearing animals (group 1A).

**Figure 1:**
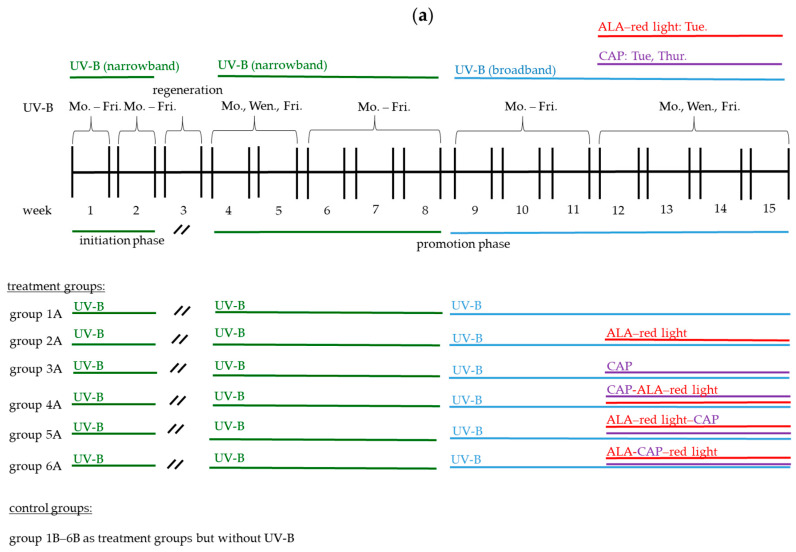

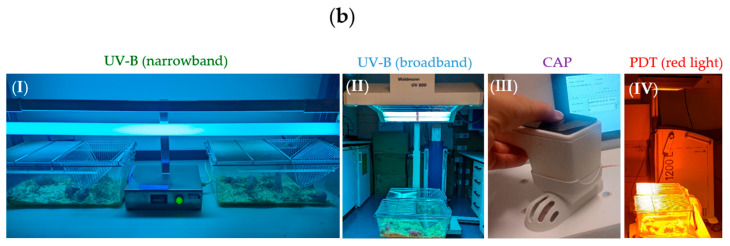
(**a**) An overview of the treatment groups (1A–6A) and the control groups (1B–6B) with the corresponding treatment regime (ALA–red light, CAP, CAP-ALA–red light, ALA–red light–CAP, ALA-CAP–red light). ALA was always incubated for 3 h before CAP or red-light treatment were added. SKH1 female mice (*n* = 9 animals per group) from the treatment groups underwent a structured UV-B treatment regimen over 15 weeks. Initially, they received narrowband UV-B treatment for eight weeks, followed by a regeneration period in week 3. Transitioning to broadband UV-B treatment started in week 9 and therapeutic intervention started in week 12. Control groups (*n* = 8 animals per group) underwent the same treatment regime but without UV-B pretreatment. (**b**) Devices used for animal treatment. UV-B treatment with narrowband UV-B (**I**) (cumulative dose of 13.724 J/cm^2^; 16 min per treatment) and broadband UV-B (**II**) (cumulative dose of 14.31 J/cm^2^; 16 min per treatment) took place in the cages (*n* = 4–5 animals per cage). CAP treatment (**III**), on the other hand, took place in a specially designed individual animal treatment chamber to ensure the targeted CAP treatment of the tumors over the short treatment time of two minutes (plasma care device; terraplasma GmbH, Garching, Germany). Hence, red-light treatment (**IV**) (100 J/cm^2^; 160 mWatt/cm^2^; 10 min; PDT-1200L, Waldmann Medizintechnik, Schwenningen, Germany) took place in the cages (*n* = 4–5 animals per cage).

An overview of all experimental groups, including control groups (1B–6B) that did not develop UV-B-induced AK or SCC, along with the treatment protocols, is presented in Figure 1a. Additionally, Figure 1b illustrates the UV-B irradiation devices, the CAP device, and the red light device used in this study.

### 2.1. Generation of UV-B-Induced Skin Tumors in SKH1 Hairless Mice

**Figure 2:**
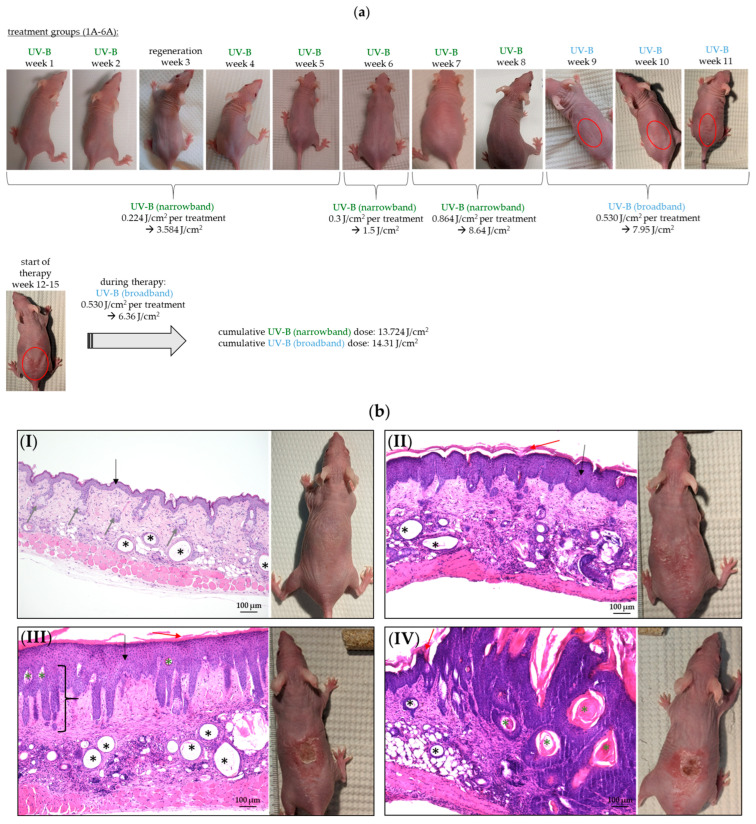
UV-B-induced AK and SCC development in SKH1 hairless mice. (**a**) SKH1 female mice (*n* = 9 animals per group) underwent a structured UV-B treatment regimen over 15 weeks. Initially, they received narrowband UV-B treatment for 8 weeks, starting with a dose of 0.224 J/cm^2^ five times a week, followed by a regeneration period in week 3. The dose was gradually increased to 0.864 J/cm^2^ at weeks 7 and 8. After transitioning to broadband UV-B treatment at a dose of 0.530 J/cm^2^, optical skin lesions appeared in 90% of the treated animals by the end of week 9. Tumor development became more pronounced at weeks 9 to 11 (red circles), leading to therapeutic intervention starting in week 12, when tumor development could be observed macroscopically in all animals; (**b**) H&E histological staining (10× objective) was conducted exemplarily at this stage (week 12) to confirm tumor manifestation. (**I**) UV-B-untreated control skin (normal skin) of SKH1 hairless mice. The skin of the mice homozygous for the hr allele exhibits characteristic features, including multiple dermal cysts (black asterisks), sebaceous gland hyperplasia (gray arrows), and the epidermis composed of a thin layer (black arrow). (**II**) Actinic keratosis displaying hyperkeratosis (red arrow) and hyperproliferation of the epidermal layer (black arrow). (**III**) SCC in situ with hyperkeratosis (red arrow), hyperproliferation showing bulbous rete with the migration of individual dysplastic cell nests into the dermis (black bracket), many cystic vacuoles (black asterisks), and small keratin pearls (green asterisks). (**IV**) Invasive SCC shows multiple dysplastic squamous cell areas with big keratin pearls (green asterisks) reaching the dermis. (**I**) Mice from the UV-B-untreated ctrl. (group 1B) and (**II**–**IV**) mice from UV-B-induced tumor development group after a cumulative UV-B (narrowband) exposure of 13.724 J/cm^2^ and a cumulative UV-B (broadband) exposure of 7.95 J/cm^2^ within 11 weeks.

In order to promote gentle tumor development without major inflammatory reaction, the treatment was initially performed with narrowband UV-B (week 1 to 8), and was conducted later with broadband UV-B (week 9 to 15). The UV-B dose was also increased over time. Tumor induction was started with the narrowband UV-B treatment phase of two weeks (initiation phase), during which the animals were irradiated five times per week with a dose of 0.224 J/cm^2^ per treatment. The animals were left untreated for regeneration in week 3, followed by repetitive narrowband UV-B treatment in weeks 4 and 5 (three times per week with the same dose of 0.224 J/cm^2^ per treatment). The UV-B dose was increased slightly in week 6 (0.3 J/cm^2^ per treatment), and the treatment interval was increased to five days per week. In weeks 7 and 8, the UV-B dose was again increased to 0.864 J/cm^2^ per treatment. After switching to broadband UV-B with a single dose of 0.530 J/cm^2^ per treatment and five treatments per week, the first skin lesions were observed in 90% of all UV-B-treated animals by the end of week 9. Tumor development became more and more pronounced during the course of the UV-B treatments from week 9 to 11 (red circles) (Figure 2a), so that the therapeutic intervention was started at week 12, when clear lesions were observed in all UV-B-treated mice. However, the observed lesions varied greatly in number, size, and depth. To confirm the tumor manifestation histologically, exemplary hematoxylin and eosin (H&E) stains were prepared at this time (Figure 2b). These were designed to transition from AK (II) to SCC (III-IV) characteristics in comparison to the normal skin of SKH1 mice (I).

### 2.2. Evaluation of Therapeutic Outcomes

After a four-week treatment with conventional ALA-PDT once a week (ALA–red light; group 2A), treatment with CAP twice a week (group 3A), or combined treatments with different treatment sequences (CAP-ALA–red light (group 4A), ALA–red light–CAP (group 5A), ALA-CAP–red light (group 6A)), employing the treatment regime described in Figure 1a, the skin lesions and their progression status were optically and histologically analyzed and compared to the untreated control group (group 1A). Additionally, the corresponding treatment groups were compared with their respective groups without UV-B treatment (groups 1B–6B) to determine whether the therapeutic intervention itself had an impact on the skin of SKH1 mice.

#### 2.2.1. Cold Atmospheric Plasma in Combination with Photodynamic Therapy Strongly Reduces the Growth of UV-B-Induced Skin Lesions

The treatment outcome for each animal was visually assessed and summarized in Table 1 according to the following criteria:

- Complete response: The lesion was eliminated.
- Good response: Reduction in lesion size was greater than 50%.
- Less response: Reduction in lesion size was 30% to 50%.
- No response: Reduction in lesion size was less than 30%.
- Progress: The lesion continued growing.

**Figure 3:**
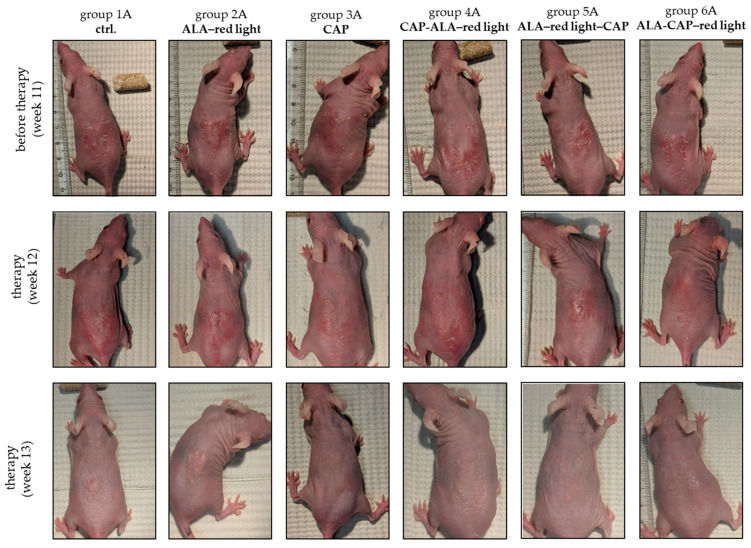

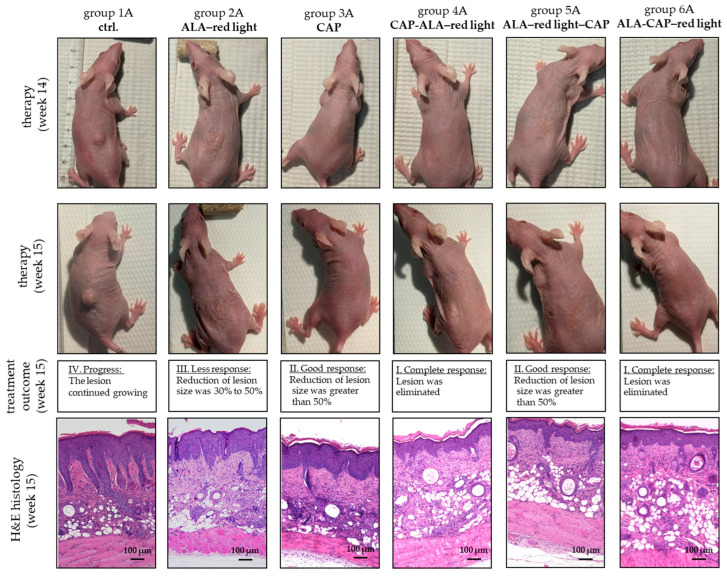
Responses of UV-B-induced skin lesions to different treatments. Representative photographs of untreated (ctrl.) animals (group 1A), ALA–red light (group 2A), CAP (group 3A), or combined treatments with different treatment sequences (CAP-ALA–red light (group 4A), ALA–red light–CAP (group 5A), and ALA-CAP–red light (group 6A)). Photographs were made just before therapy (week 11) and once per week during the four weeks of therapy (week 12 to 15). The animals were killed after therapy, and the affected skin areas were processed for histological and molecular biological examination. H&E histology shows the status quo of the skin after therapy (10× objective). The treatment success depended on the treatment regimen and was improved in every animal except the animals of the untreated ctrl. (group 1A).

Visual examination and digital photographs (once per week) documented lesion multiplicity before and after the treatments (Figure 3).

#### 2.2.2. Cold Atmospheric Plasma in Combination with Photodynamic Therapy Reduces the Epidermal Thickness

**Figure 4:**
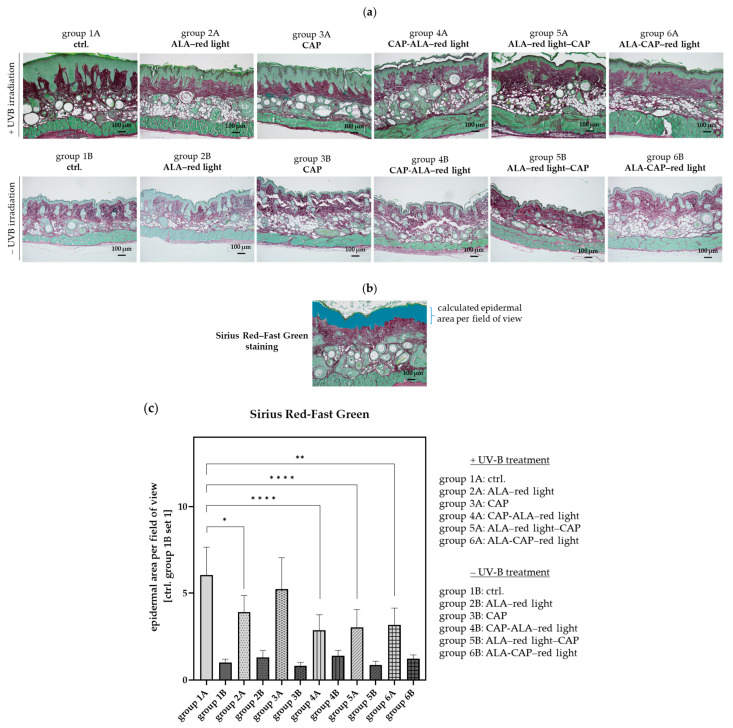
The measurement of the epidermal thickness in UV-B-induced skin lesions and non-irradiated skin after different therapeutic interventions. (**a**) Sirius Red-Fast Green-stained paraffin sections of UV-B-induced tumor mice (groups 1A–6A) and UV-B non-irradiated mice (groups 1B–6B) after different treatments (ALA–red light, CAP, CAP-ALA–red light, ALA–red light–CAP, ALA-CAP–red light). (**b**) The area of the epidermis per field of view (10× objective) was calculated with the BZ-H4C software (Version 1.1.2.4, Keyence, Neu-Isenburg, Germany). (**c**) The epidermal area correlates with the epidermal thickness and is calculated for all animals (*n* = 7–8) per group, employing ctrl. group 1B set 1. Statistical analysis: Ordinary one-way ANOVA with Bonferroni’s multiple comparison test was performed to compare the mean of group 1A with the corresponding treatment groups (2A–6A). * *p* ≤ 0.05, ** *p* < 0.01, **** *p* < 0.0001.

In contrast to the control group without UV-B irradiation (group 1B), UV-B irradiation greatly increases epidermal thickness (group 1A) (Figure 4a,c). Epidermal thickness was calculated by measuring the epidermal area per field of view (10× objective), as shown in Figure 4b.

Conventional ALA–red-light treatment (group 2A) significantly decreased epidermal thickness. An even greater reduction in epidermal thickness was observed after the combined treatments (CAP-ALA–red light, ALA–red light–CAP, ALA-CAP–red light) (Figure 4a,c). CAP treatment alone (group 3A) appeared to have no significant impact on epidermal thickness under the treatment conditions used, although clear visual improvement of the skin can be seen in Figure 3. With normal skin, the different treatments (groups 1B–6B) had no significant effect on epidermal thickness (Figure 4a,c).

#### 2.2.3. Cold Atmospheric Plasma in Combination with Photodynamic Therapy Reduces Proliferation of Keratinocytes in Affected Skin Areas

**Figure 5:**
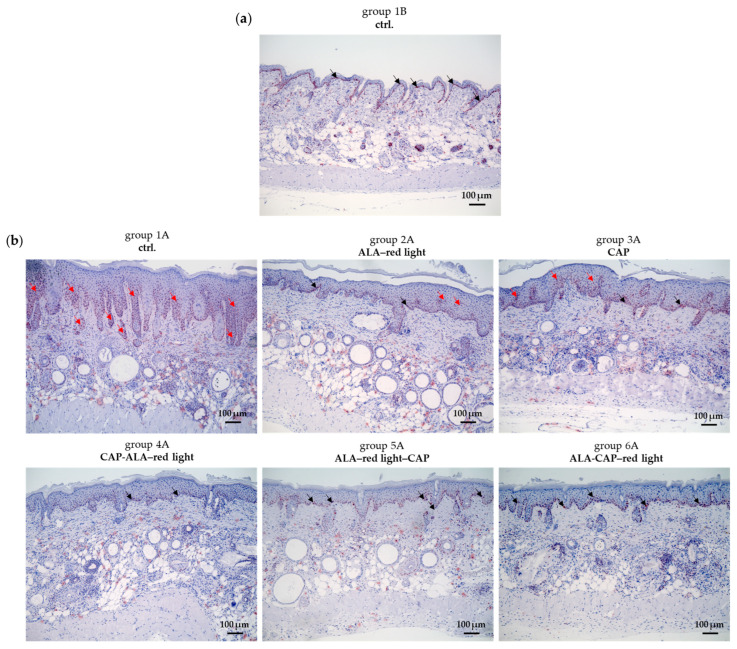

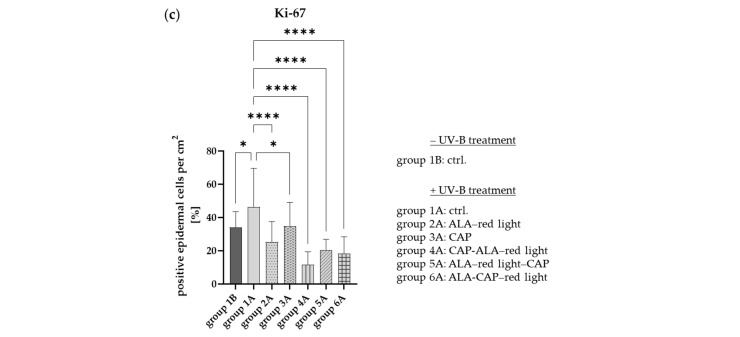
Ki-67 immunostaining of skin tissue sections. (**a**) Ki-67 expression of proliferating kerationcytes of basal cell layer (black arrows) in normal skin of SKH1 hairless mice. (**b**) Patched pattern of Ki-67 expression (red arrows) can be detected in UV-B-induced skin lesions without therapy (group 1A) and strong reduction in Ki-67 positive cells can be observed after different therapeutic interventions (groups 2A–6A). (**c**) Percentage of positive cells in epidermis per square centimeter was measured across three representative regions per image (from a sample of 7 to 8 animals per group) at 10× magnification. Statistical analysis: Ordinary one-way ANOVA with Bonferroni’s multiple comparison test was performed to compare the mean of ctrl. (group 1A) with all treatment groups (groups 2A–6A) and group 1B. * *p* ≤ 0.05, **** *p* < 0.0001.

The proliferation marker Ki-67 is typically found in healthy epidermis, predominantly within the basal cell layer, as illustrated in the control skin samples from group 1B (Figure 5a; black arrows) and summarized in Figure 5c. In contrast, Ki-67 expression is significantly heightened in UV-B-induced skin lesions (Figure 5b,c; group 1A), being particularly concentrated in the rete (red arrows), as well as within dysplastic cell nests. After ALA–red-light treatment (group 2A) and CAP treatment (group 3A), Ki-67 expression was diminished; however, in both cases, proliferating cells were still observed within the basal cell layer (black arrows) and in the upper layers of the epidermis (stratum spinosum and stratum granulosum), as indicated by the red arrows. After the combined treatments (groups 4A–6A), there was a notable decrease in Ki-67 expression, with proliferation primarily restricted to the basal cell layer, like the ctrl. group 1B. These combined therapies may generate excess amounts of ROS that damage cellular components, leading to reduced cellular proliferation and the normalization of epidermal growth patterns, thereby limiting primarily hyperproliferation to the basal layer.

#### 2.2.4. Cold Atmospheric Plasma in Combination with Photodynamic Therapy Induces Apoptosis in Affected Skin Areas

**Figure 6:**
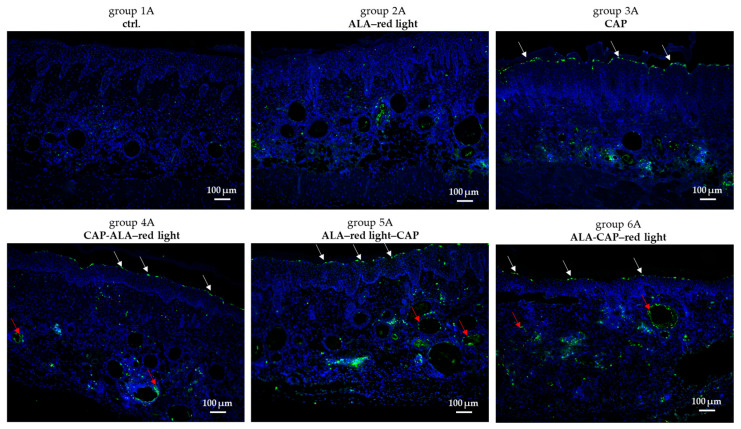
In situ TUNEL staining of tissue sections. Measurement of apoptosis in UV-B-induced skin lesions after different therapeutic interventions (ALA–red light, CAP, CAP-ALA–red light, ALA–red light–CAP, ALA-CAP–red light) and in untreated ctrl. animals are shown exemplarily. White arrows show apoptotic cells (green fluorescence staining) of stratum granulosum of epidermis. Red arrows show apoptotic cells (green fluorescence staining) in epithelial lining of cyst wall. Blue color shows DAPI staining in nucleus.

In control tissue sections (group 1A), only a limited number of apoptotic cells, indicated by green fluorescence staining, were observed compared to the treatment groups (groups 2A–6A) (Figure 6). Notably, CAP treatment resulted in cell apoptosis in the stratum granulosum of the epidermis (white arrows), a phenomenon that was also evident after combined treatment (groups 4A–6A). In contrast, ALA–red light treatment alone (group 2A) did not exhibit this characteristic. Additionally, a significant increase in apoptosis was noted in the epithelial lining of the cyst wall (red arrows) after combined treatment (groups 4A–6A).

#### 2.2.5. Cold Atmospheric Plasma in Combination with Photodynamic Therapy Reduces DNA Damage in Affected Skin Areas

**Figure 7:**
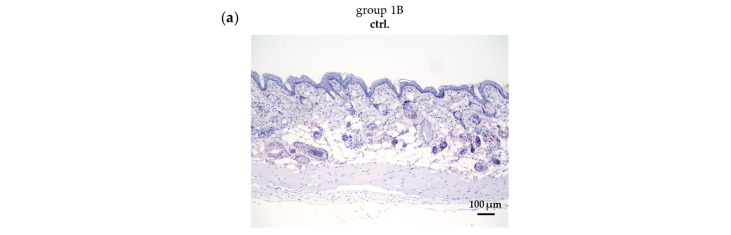

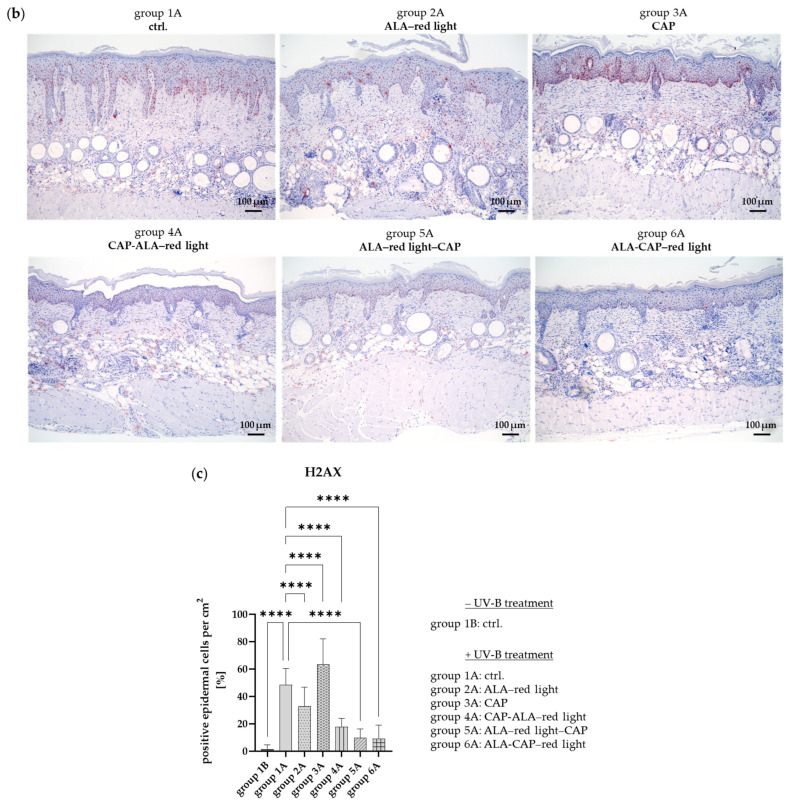
γH2AX immunostaining of skin tissue sections. (**a**) γH2AX, marker for DNA double-strand breaks (DSBs) from ionizing radiation, is not expressed in normal skin of SKH1 hairless mice. (**b**) Strong expression pattern of γH2AX was detected in UV-B-induced skin lesions (group 1A). Significantly fewer positive cells were observed in epidermis after ALA–red-light treatment (group 2A), whereas after CAP treatment, increase in γH2AX positive cells was detected compared to untreated control (group 1A). After double therapy, however, there was strong reduction in γH2AX expression in all treatment sequences applied (groups 4A–6A). Photos were taken with 10× objective. (**c**) Percentage of positive cells in epidermis per square centimeter was measured across three representative regions per image (from a sample of 7 to 8 animals per group) at 10× magnification. Statistical analysis: Ordinary one-way ANOVA with Bonferroni’s multiple comparison test was performed to compare mean of ctrl. (group 1A) with all treatment groups (groups 2A–6A) and group 1B. **** *p* < 0.0001.

We analyzed the expression of γH2AX in normal skin and in UV-B-induced skin lesions with and without therapy; as expected, γH2AX is not detectable in the normal skin of SKH1 hairless mice (Figure 7a,c). A strong expression pattern of γH2AX was observed in UV-B-induced skin lesions (group 1A) (Figure 7b,c). After ALA–red-light treatment (group 2A), significantly fewer positive cells were detected in the epidermis, whereas after CAP treatment, there was an increase in γH2AX-positive cells compared to the untreated control group (Figure 7b,c). Hence, after double therapy, a marked reduction in γH2AX expression was noted across all treatment groups (groups 4A–6A) (Figure 7b,c).

### 2.3. Evaluation of Molecular Changes

#### 2.3.1. Cold Atmospheric Plasma in Combination with Photodynamic Therapy Induces Anti-Tumor Immune Responses

**Figure 8:**
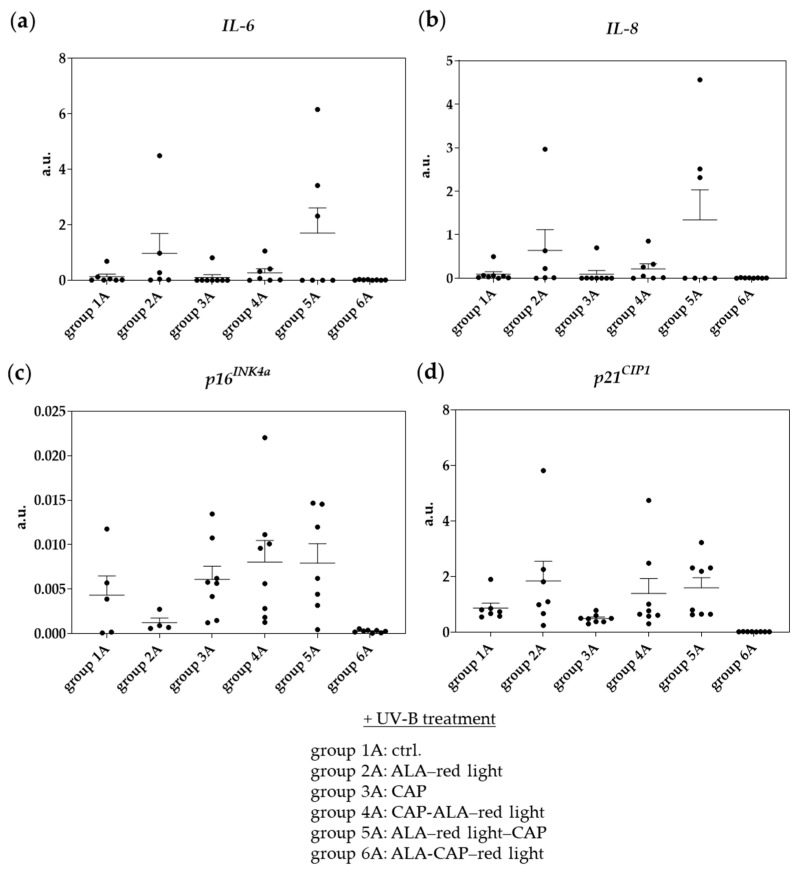
Expression of cytokines and apoptosis-related molecules on mRNA level after different treatments (groups 2A–6A) and in untreated ctrl. skin (group 1A). (**a**,**b**) mRNA expression of cytokines (*IL-6*, *IL-8*) and (**c**,**d**) apoptosis-related molecules (*p16^INK4a^*, *p21^CIP1^*) isolated from full skin (epidermis and dermis) preparations. Expression intensity was indicated in arbitrary units (a.u.). Statistical analysis: Ordinary one-way ANOVA with Bonferroni’s multiple comparison test was performed to compare mean of ctrl. (group 1A) with all treatment groups (groups 2A–6A). Significant results were not detected.

Interleukin-6 (IL-6) and interleukin-8 (IL-8) are cytokines that play crucial roles in immune responses, inflammation, and tumor biology [32,33,34,35]. In our study, we examined the expression levels of these cytokines in affected skin regions (epidermis and dermis) after therapeutic intervention and compared them to untreated control skin (group 1A). Although we did not find any significant differences in expression levels due to the considerable variability among individual mice within each group, we observed an increase in both *IL-6* (Figure 8a) and *IL-8* (Figure 8b) levels after treatment with ALA–red light (group 2A). Furthermore, the combined treatment of ALA–red light followed by CAP (group 5A) led to an even greater increase in the expression of both cytokines.

#### 2.3.2. Cold Atmospheric Plasma in Combination with Photodynamic Therapy Induces Apoptosis-Related Molecules

The expression of *p16^INK4a^* and *p21^CIP1^* in SCC after treatments such as PDT or CAP can provide insights into the cellular response to these therapies. ALA–red light (group 2A) reduced the expression of *p16^INK4a^*, whereby *p21^CIP1^* appeared to increase in comparison to the untreated control (Figure 8c,d). CAP treatment and treatment with double therapy with the sequence CAP-ALA–red light and ALA–red light–CAP (groups 4A and 5A) induced *p16^INK4a^* in most of the animals examined. Interestingly, the gene expression of *IL-6*, *IL8*, *p16^INK4a^*, *and p21^CIP1^* was virtually undetectable after double therapy with the ALA-CAP–red light sequence (group 6A). This could imply that this combined treatment effectively suppresses inflammatory responses and cell cycle regulators at the gene expression level, possibly indicating a more profound therapeutic effect or the suppression of tumor-promoting pathways.

## 3. Discussion

PDT and CAP are two treatment modalities. Both have shown promise in the management of skin conditions, such as AK or initial SCC [21,36,37,38].

PDT involves the application of a photosensitizing agent that is activated by specific wavelengths of light. This activation leads to the production of ROS that cause cellular damage and apoptosis in targeted cancerous or precancerous cells, while sparing surrounding healthy tissue [39]. The effectiveness of PDT is limited in deeper lesions. However, numerous reports suggest that topical PDT can produce favorable outcomes in the treatment of superficial and microinvasive SCC and is particularly suitable for patients or lesion sites that cannot tolerate surgery [11,12,14,15]. The effectiveness of PDT is limited by factors such as the depth of light and sensitizer penetration, the dosage of the photosensitizing agent and light, and the volume of the lesions to be treated. Therefore, it is important to find alternative methods to optimize treatment outcome.

CAP generates a partially ionized gas that contains reactive oxygen and nitrogen species (RONS), including ions, electrons, and neutral particles [40]. These components can induce oxidative stress in cells, leading to apoptosis and the necrosis of abnormal cells [41]. CAP has been explored for various dermatological conditions [42,43]. Its effectiveness on SCC and AK has been demonstrated [17,18] but is still under investigation, and long-term efficacy data are limited. However, its non-invasive nature and painless application make it an attractive option for patients who cannot tolerate more aggressive treatments. In addition to its selective anti-tumorigenic property, CAP has the ability to temporarily destabilize the skin barrier. This way, the transdermal administration of substances (such as ALA) can be improved with a mechanism similar to electroporation [23,25,26,27,44].

Different strategies have been tried to improve the efficacy of PDT using pharmacologic, keratolytic, or physical pretreatments to increase PpIX accumulation in the tumor or to induce additional anti-carcinogenic effects. Several modifications of ALA (Methyl-ALA, Hexyl-ALA, ALA-Esters, N-Ac-ALA, ALA in liposomes or nanoparticles) have been made to improve cellular uptake and to increase the efficacy of PDT. In addition, CAP can influence the clinical outcome of PDT by affecting the permeability of cell membranes and combining this ability with its anti-tumor properties. This dual function, which improves the therapeutic efficacy of PDT, is a unique advantage of CAP, which is why it is important to clarify this mechanism.

The aim of this study was to use the permeability-enhancing properties together with the anti-tumorigenic properties of CAP [38,45,46,47] to increase the efficacy of conventional PDT for the treatment of AK and SCC in vivo.

The efficacy of this combined treatment has already been analyzed in vitro on different SCC cell lines of cutaneous and oral origin and this showed an increase in anti-tumor efficacy [31]. This in vitro study showed that the application sequence of CAP and ALA prior to red light illumination (either CAP before ALA incubation for 3 h or CAP after 3 h of ALA incubation) significantly contributes to the success of the therapy. The ALA-CAP–red light sequence achieved much better results, most likely by exceeding the lethal threshold of ROS for tumor cells [31].

The extent to which this combined treatment enhances the therapeutic effect in vivo has not been investigated so far. This was carefully examined in the present study on UV-B-induced skin tumors in hairless mice. When interpreting the results, the sequence of the treatments (CAP-ALA–red light, ALA–red light–CAP or ALA-CAP–red light) was also taken into account for the future clinical use of this therapy.

First, the focus was on the therapeutic outcomes and parameters such as tumor growth reduction, epidermal thickness, cell proliferation, apoptosis, and DNA damage. Only after assessing these factors were molecular changes evaluated in a subsequent step. Untreated control animals (group 1A) were compared with animals treated either monotherapeutically with conventional ALA-PDT (group 2A) or CAP (group 3A) or treated with both therapies combined in different treatment sequences (groups 4A–6A). The corresponding treatment groups were compared with their respective groups without UV-B treatment (groups 1B–6B) to determine whether the therapeutic intervention itself had an impact on the skin of SKH1 mice. As expected and also shown in other studies [16,48,49], histopathological changes could not be detected in normal skin (Figure 4a,c), which confirms the selectivity of both therapies against tumor cells.

Tumor growth reduction in UV-B-induced skin tumors was achieved after therapeutic intervention with varying degrees of success (Table 1; Figure 3). Indeed, 62.5% of animals treated with ALA-PDT (group 2A) showed a good treatment response, with no animal in complete remission. The study by Wang et al. (2013) showed similar results, as they achieved complete tumor remission after four ALA-PDT sessions (8% ALA, 3 h incubation, 30 J/cm^2^ at 20 mW/cm^2^) in only 84% of very small SCC lesions (1–4 mm); tumor remission in large and thick SCC (>5 mm and thickness >2.5 mm) only showed <50% reduction [50]. CAP treatment in our study resulted in complete response in 28.6% of patients, in a good response in 57.1% of patients, and in a lesser response in 14.3% of patients. Several reports have described the decline in SCC growth after CAP treatment [20,38,51,52]. Pasqual-Melo and colleagues showed for the first time that CAP treatment also limits the development of endogenously generated, UV-B-induced skin lesions with characteristics of SCC [16].

After the combined application, an improved treatment success was recorded macroscopically, particularly in groups 4A and 6A (Table 1; Figure 3). However, when the microscopic analyses (epidermal area, proliferation, apoptosis, DNA damage) were included in the evaluation, no preference was found for the sequence of application. In vitro, the sequence of application entailing the use of ALA-CAP–red light on SCC cells seems to be more efficacious than that using CAP-ALA–red light. This increased efficacy is probably due to higher intracellular ROS levels when ALA is applied first, followed by CAP and red-light treatment [31]. These differences between in vitro and in vivo observations may be attributed to the involvement of paracrine mechanisms (communication between different cell types), which are not accounted for in vitro monoculture conditions. In vivo, however, factors such as tissue perfusion, immune response, and tumor microenvironment play a crucial role. The prior application of CAP may potentially modify tumor tissue, making it more sensitive to PDT. Alternatively, initial PDT treatment may damage the tissue in such a way as to make CAP more effective afterwards by killing remaining cells or by activating the immune system. Further studies are essential to understand the cellular and molecular mechanisms that are triggered when conventional PDT is used in combination with CAP in order to specifically promote therapeutic efficacy.

After UV-B treatment, H2AX phosphorylation is a key marker for detecting DNA damage and for initiating repair mechanisms within the cell [53]. This process is critical for maintaining genomic stability and for preventing mutations that could lead to cancer or other diseases. In the present study, the histological γH2AX staining (Figure 7) revealed interesting results. As expected, strong DNA damage was induced by UV-B radiation in untreated animals (group 1A), whereas conventional PDT treatment (group 2A) led to a significant decrease in γH2AX staining. The level and persistence of γH2AX expression after PDT can correlate with treatment efficacy. A robust γH2AX response may indicate significant DNA damage and could be associated with effective tumor cell killing. Conversely, if SCC cells exhibit a strong ability to repair this damage quickly, this may lead to treatment resistance. CAP treatment (group 3A), however, appeared to further increase γH2AX expression compared to the untreated control. Increased H2AX phosphorylation was also observed in a study involving kINPen plasma treatment; according to the authors, this did not seem to be due to ROS-mediated DNA damage, but rather a consequence of CAP-induced apoptosis. Furthermore, the authors could not identify any lasting mutagenic effects from CAP treatment [54]. Surprisingly, all conducted combinations of CAP and PDT treatments in the present study showed significantly reduced γH2AX expression.

Mechanistically, the initial increase in γH2AX following CAP treatment may be attributed to the induction of apoptosis pathways that activate DNA damage signaling cascades. ROS generated by CAP can cause oxidative modifications of DNA bases and strand breaks, leading to H2AX phosphorylation as part of the cellular response to DNA damage. However, the subsequent reduction in γH2AX levels observed when combining CAP with PDT suggests that these treatments may modulate cellular responses differently—potentially through enhanced apoptotic clearance of damaged cells or the activation of repair mechanisms that limit persistent DNA damage signals.

Further research is needed to better understand these underlying mechanisms, particularly how CAP influences DNA repair pathways and apoptotic signaling when combined with PDT. Such insights could help to optimize combination therapies for improved tumor control while minimizing potential genotoxic side effects.

Interleukin-6 (IL-6) and interleukin-8 (IL-8) are cytokines that play significant roles in immune response, inflammation, and tumor biology [32,33,34]. In the context of SCC, their expression and function can be rather complex, especially after treatments such as PDT or CAP. While IL-6 can have pro-tumor effects, it also has a role in modulating anti-tumor immunity [55,56]. Similar to IL-6, IL-8 can have dual roles; while it recruits immune cells to the tumor microenvironment, it may also contribute to an immunosuppressive environment that allows tumors to evade immune detection. In the present work, we observed an increase in both cytokines after ALA–red-light treatment (group 2A; Figure 8a,b) and a further increase after double treatment with the treatment sequence ALA–red light–CAP (group 5A), suggesting the induction of anti-tumor immune responses as already described by Wang et al. (2013) for conventional PDT treatment [50]. However, these mRNA expression data should not be over-interpreted because the results do not appear significant due to the large variance between the animals within a treatment group. Understanding how the combined treatment of PDT and CAP affects cytokine mRNA expression levels such as *IL-6* and *IL-8* in SCC may help to optimize treatment protocols and improve therapeutic outcomes by balancing anti-tumor effects with potential inflammatory responses.

To gain more insights into the cellular response to these therapies, we analyzed the expression of *p16^INK4a^* and *p21^CIP1^* in SCC after treatments. Changes in *p16^INK4a^* or *p21^CIP1^* expression may indicate alterations in cell cycle regulation, senescence, and the apoptosis of tumor cells [57,58]. Increased expression may reflect cellular damage, leading to growth arrest and possibly enhanced apoptosis. With a few exceptions, the therapeutic treatment of AK and SCC animals tends to increase the expression of *p16^INK4a^* and *p21^CIP1^* (Figure 8c,d), which correlates with the induction of apoptosis observed by the TUNEL assay (Figure 6). Overall, no significant differences were observed between treatment groups, indicating that these therapies do not produce consistent or dramatic changes in these markers across all samples. However, the variability highlights the importance of further studies to understand individual responses and optimize treatment protocols.

## 4. Materials and Methods

### 4.1. Animals

Female SKH1 mice (4–8 weeks old; #686SKH1-HR; Charles River Laboratories, Sulz-feld, Germany) were maintained under specific pathogen-free and controlled conditions (22 °C, 55% humidity, and 12 h day/night rhythm) and had free access to water and chow. The mice received humane care in compliance with the guidelines outlined in the Guide for the Care and Use of Laboratory Animals. The study was approved by the Research Ethics Committee (approval number: 55.2.2.-2532-2-1608-38) from the government of Lower Franconia, Würzburg, Germany. All animals (*n* = 102) were divided into experimental groups (*n* = 9 mice per group; 1A–6A) and control groups (*n* = 8 mice per group; 1B–6B). After a week of acclimatization in the group cages (*n* = 4–5 animals per cage), UV-B-induced tumor development was started using the treatment regimen shown in Figure 1a.

### 4.2. UV-B-Induced Animal Model of Actinic Keratosis and Squamous Cell Carcinoma

From an animal welfare perspective and to improve the reproducibility of tumor induction, we aimed to achieve as “gentle” a tumor induction as possible in our experimental approach. Therefore, we conducted an initial promotion phase using narrowband UV-B to first induce UV adaptation. By limiting the wavelength range to a narrower spectrum (305–315 nm), unwanted side effects such as inflammatory responses or skin damage are minimized.

However, in the natural environment and in therapeutic applications (e.g., light therapy), broadband sources are often used. The combination of narrowband and broadband UV-B thus allows for a better translation of the results to clinical or environmental conditions, which is why we chose to use a combination of both spectra in this study.

To induce skin transformation, animals from groups 1A–6A were exposed to UV irradiation using a Philips UV-B Narrowband TL lamp (TL 40W/01 RS with narrow waveband of between 305 and 315 nm peaking at 311 nm; Philips Lighting, Distrelec Deutschland GmbH, Bremen, Germany) and a Philips UV-B Broadband TL lamp (TL 40W/12 with broad waveband of between 290 and 315 nm; Svetila.com; Domzale, Slovenia).

The UV dose was measured with a UV detector (VarioControl, Waldmann, Villingen-Schwenningen, Germany) that was calibrated for the respective UV-B spectral range yielding a readout in J/cm^2^. UV-B treatment took place in the respective group cages as shown in Figure 1b(I,II). Therefore, we removed enrichment and food in the cages, except for the bedding, to prevent the animals from hiding during the treatment.

The treatment regime was based on the study by Pasqual-Melo et al. (2020) [16]. The respective UV dose, including the total dose of narrowband and broadband UV-B, was further developed in the present study and can be seen in Figure 2a. The first macroscopic changes were visible after week 8 (Figure 2a). From this point onwards, treatment was switched to broadband UV-B irradiation. Visible AK or SCC tumors were recorded after week 11, so that the therapeutic intervention was carried out from week 12 to 15. During this time, UV-B irradiation was continued three times per week (broadband UV-B; 0.530 J/cm^2^ per treatment) to avoid spontaneous tumor remission due to UV-B discontinuation. The extent of tumor development (groups 1A–6A) was examined exemplarily (H&E staining) in one animal per group before the start of therapy (Figure 2b).

### 4.3. Plasma Device

A prototype of the plasma care^®^ device, developed by terraplasma GmbH, Garching, Germany, was used for CAP treatment. A recent publication detailed the design, technology, and ozone emission spectrum of this device [59]. This prototype enables frequency adjustments between an oxygen mode (4 kHz) and a nitrogen mode (8 kHz), with a 4 kHz setting specifically used in this study. The device employs a technology known as “thin-film technology”, which is an advanced version of surface micro-discharge technology (SMD) [60]. The application of a high voltage of 3.5 kV generates millimeter-sized micro-discharges within the plasma-source unit. The unit consists of a high-voltage electrode, a dielectric, and a grounded structured electrode [59], which together produce plasma components that can be adjusted based on frequency and voltage.

### 4.4. Treatment of Mice with Cold Atmospheric Plasma

Mice were treated with CAP from week 12 to 15, twice a week on Tuesdays and Thursdays (plasma care^®^ device: 2 min per treatment, 4 kHz; Figure 1a), while PDT was performed with ALA and red light only once a week on Tuesdays in combination with CAP treatment. The treatment area of the plasma electrode was only 2.5 × 2.5 cm, so treatment of a single animal was chosen. For dorsal treatment, the animals were individually placed under the plasma source in a 3D-printed treatment tube (custom-made with an adapter for the plasma care^®^ device by terraplasma GmbH, Garching, Germany) (Figure 1b(III)). After each CAP treatment, the animals were returned to their housing group cages.

### 4.5. Treatment of Mice with 5-Aminolaevulinic Acid (ALA)

Based on the principle of pain reduction and the principle of lowest possible ALA dose [50], 8% ALA-HCL was prepared in Neribas ointment (pharmacy at the UKR Regensburg). The ointment was generously applied (~1 mm thick) to the dorsal skin of the back using a cotton pad. In control animals that did not develop lesions (groups 2B, 4B, 5B, 6B), the cream was applied to the entire back. For UV-B-induced tumor mice (groups 2A, 4A, 5A, 6A), particular attention was paid to ensure that all lesion sites were well covered with cream. The sequence of the ALA treatment depended on the corresponding treatment group. In groups 2A and 2B, ALA was applied for 3 h before red-light treatment. In groups 4A and 4B, incubation with ALA was carried out immediately after the 2 min CAP pretreatment. In groups 5A and 5B, ALA was applied 3 h before illumination with red light, immediately followed by CAP treatment. In groups 6A and 6B, ALA was applied 3 h before CAP treatment, immediately followed by red light irradiation. During the 3 h ALA incubation, the cages were kept in the dark. Before red light irradiation or CAP treatment, excess ALA was carefully removed with a paper towel.

### 4.6. Light Source and Red Light Irradiation of Mice

The mice were irradiated with red light (Figure 1b(IV)) using an incoherent light source (λ_em_ = 575–750 nm, PDT-1200L, Waldmann Medizintechnik, Schwenningen, Germany) with a light intensity of 160 mW/cm^2^ (100 J/cm^2^). Irradiation (~10 min per treatment) took place in the group cages without enrichment and food during the treatment. For the conventional ALA-PDT (groups 2A, 2B), the red-light treatment was carried out after ALA incubation for 3 h. In the combined CAP-ALA–red light application groups 4A and 4B, the mice were treated with CAP for 2 min (see Section 4.4). Immediately after CAP treatment, the animals were incubated with ALA for three hours (see Section 4.5) and immediately thereafter treated with red light. In the ALA–red light–CAP application groups 5A and 5B, the animals were incubated with ALA for 3 h (see Section 4.5), followed by red light irradiation (see above) and CAP treatment (see Section 4.4) immediately thereafter. In the ALA-CAP–red light application groups 6A and 6B, animals were incubated with ALA for 3 h and treated with CAP for two minutes. Immediately after CAP treatment, mice were treated with red light as described above.

It should be mentioned that immediately after red-light treatment, we observed nervous behavior, pronounced redness, and scratching and licking of the affected skin areas. These skin changes lasted up to a maximum of 24 h. Edema and scabbing, as reported by Wang et al. (2013) [50], were not observed in the current study.

### 4.7. Macroscopic Evaluation of Therapeutic Outcomes

During tumor induction and the treatment phases, the animals were monitored daily and digitally photographed once a week (Fridays). The initial measurements of tumor size and depth prior to the initiation of therapy were determined with limited accuracy and were not included in the evaluation. All UV-B-treated animals exhibited tumors of varying appearance and size prior to the initiation of therapy, leading to their inclusion in the treatment groups. After the last treatment session, the animals were euthanized the next day by cervical dislocation, and the affected skin areas were promptly excised for histological and molecular analyses. The treatment outcome for each animal was assessed visually and summarized in Table 1 according to the following criteria: I. Complete response: The lesion was entirely eradicated. II. Good response: The lesion size decreased by more than 50%. III. Partial response: The lesion size decreased by 30% to 50%. IV. No response: The lesion size decreased by less than 30%. V. Progress: The lesion continued growing.

### 4.8. Histological Evaluation of UV-B-Induced Tumor Development

Histological examination was conducted on formalin-fixed and paraffin-embedded full-skin preparations from all mice (*n* = 102). Skin sections measuring 2 µm were stained with hematoxylin and eosin (H&E) and Sirius Red-Fast Green to examine extracellular matrix accumulation and the thickening of the epidermal structure. Both stains were applied according to the procedure described elsewhere [61].

### 4.9. Evaluation of the Epidermal Thickness

Epidermal thickness was measured using tissue sections stained with Sirius Red-Fast Green (see Section 4.8) and photographed with the All-in-One Microscope BZ-X800 (Keyence, Neu-Isenburg, Germany) and the analysis software BZ-H4C (Version 1.1.2.4, Keyence, Neu-Isenburg, Germany). All calculated epidermal areas were checked and corrected manually, if necessary. The area of the epidermis was calculated per field of view (10× objective) and is shown exemplarily in Figure 4b. The epidermal area correlates with epidermal thickness and is calculated for all animals (*n* = 7–8) per group with ctrl. group 1B set to 1.

### 4.10. Histological Evaluation of Proliferation and DNA Damage

Immunohistological staining was performed on 2 μm formalin-fixed, paraffin-embedded full-skin sections. To analyze proliferation, antigen retrieval was performed with Tris-EDTA buffer (1 mM, pH 8.0) at 96 °C for 30 min using a steamer. Prior to the incubation with the primary antibody, non-specific binding was minimized by applying a blocking solution (rabbit Superblock; Zytomed Systems GmbH, Berlin, Germany) for 5 min. The Ki-67 [SP6] antibody (Abcam, Cambridge, UK) was then applied at a dilution of 1:500 and incubated for 60 min at room temperature. The Histofine Simple Stain MAX PO (anti-rabbit) universal immuno-peroxidase polymer detection system (Medac Diagnostica, Wedel, Germany) was used according to the manufacturer’s specifications. Positive staining was visualized using 3-Amino-9-Ethylcarbazole (AEC) (Zytomed), and the samples were counterstained with hematoxylin (Merk, Darmstadt, Germany). To assess DNA damage, antigen retrieval was performed on tissue sections using a citrate buffer (pH 6.0) in a steamer at 96 °C for 30 min. Prior to the incubation with the primary antibody, non-specific binding was blocked by applying a blocking solution (rabbit Superblock; Zytomed Systems GmbH) for 5 min. The Phospho-Histone H2AX (Ser139) (20E3) antibody (1:500; Cell Signaling, Frankfurt, Germany) was then incubated overnight at 4 °C. The sections were then treated with Histofine Simple Stain MAX PO (anti-rabbit) for 30 min. After washing, positive reactions were visualized with AEC (Zytomed), and the samples were subsequently counterstained with hematoxylin (Merk). Both stainings were evaluated semi-quantitatively by means of light microscopy (All-in-One Microscope BZ-X800; Keyence, Neu-Isenburg, Germany). For scoring, the number of positive cells in the epidermis per square centimeter was measured across three representative regions per image (from a sample of 7 to 8 animals per group) at 10× magnification. These results were then compared to those of the control group (group 1A).

### 4.11. Histological Evaluation of Apoptosis

The terminal deoxynucleotidyl transferase (TdT) dUTP Nick-End Labeling (TUNEL) assay was used to detect apoptotic cells that underwent extensive DNA degradation during the late stages of apoptosis. The DeadEnd™ Fluorometric TUNEL System (Promega GmbH, Walldorf, Germany) was performed on 2 μm formalin-fixed, paraffin-embedded full-skin sections according to the manufacturer’s instructions, and the staining was evaluated by fluorescence microscopy (All-in-One Microscope BZ-X800; Keyence, Neu-Isenburg, Germany).

### 4.12. Isolation of Ribonucleic Acid (RNA) and Reverse Transcription

RNA from full-skin tissue was isolated immediately after tissue asservation using the NucleoSpin^®^ RNA Plus Kit (Macherey-Nagel, Düren, Germany) according to the manufacturer’s instructions. cDNA was generated with the SuperScript II Reverse Transcriptase kit (Invitrogen; Thermo Fisher Scientific, Waltham, MA, USA) using 2–5 µg of total RNA for transcription according to the manufacturer’s instructions.

### 4.13. Quantitative Real-Time Polymerase Chain Reaction (PCR) Analysis

Gene expression analysis was conducted using quantitative real-time PCR with specific primer sets (Sigma-Aldrich, Steinheim, Germany) and conditions outlined in Table 2. The analysis was carried out using LightCycler technology (Roche Diagnostics, Mannheim, Germany) according to the procedures described in previous studies [62]. PCRs were assessed through melting curve analysis. To confirm the integrity of cDNA and to normalize expression levels, beta-actin (β-actin) was amplified. Each experiment was conducted at least three times, with each run performed in duplicate. For the graphical representation, a dot plot was chosen, where each displayed point represents the results of an individual animal. *n* = 8 animals were examined per group; if fewer than 8 points were recognizable, then the expression values were out of range <0.

### 4.14. Statistical Analysis

All data were analyzed using GraphPad Prism 10.2.2 software (GraphPad Software Inc., San Diego, CA, USA) and are presented as mean values ± standard deviation (SD). To determine the significant differences among the various groups, ordinary one-way ANOVA was performed, followed by Bonferroni’s multiple comparison tests. Significant results are indicated * *p* ≤ 0.05, ** *p* < 0.01, *** *p* < 0.001, or **** *p* < 0.0001. A detailed description of the statistics is given in the corresponding legend below the respective figure.

## 5. Conclusions

CAP represents a promising advancement in the treatment of actinic keratosis and squamous cell carcinoma, with the potential to enhance the efficacy of conventional therapies such as photodynamic therapy with 5-aminolevulinic acid. This preclinical study indicates that the combination of CAP with ALA-PDT may improve anti-tumor effects in UV-B-induced tumors in hairless mice. However, in contrast to in vitro findings, the sequence of application does not appear to significantly impact treatment outcomes in vivo. Further research is necessary to evaluate the efficacy and optimal application strategies of this combined treatment approach in human patients.

## Figures and Tables

**Table 1:** Visual examination of lesion multiplicity after four weeks of treatment.

Treatment Group	Treatment	Treatment Outcome [%]				
*n* = (7–8)		I. ^1^	II. ^1^	III. ^1^	IV. ^1^	V. ^1^
1A	ctrl.	-	-	-	-	100
2A	ALA–red light	-	62.5	37.5	-	-
3A	CAP	28.6	57.1	14.3	-	-
4A	CAP-ALA–red light	50.0	37.5	12.5	-	-
5A	ALA–red light–CAP	14.3	71.5	14.3	-	-
6A	ALA-CAP–red light	50.0	37.5	12.5	-	-

^1^ Categorization of treatment outcome: I. complete response; II. good response; III. less response; IV. no response; V. progress.

**Table 2:** Murine primers and conditions.

Primer Name	Forward Primer 5′ → 3′	Reverse Primer 5′ → 3′	Condition ^1^
β-actin	TGGAATCCTGTGGCATCCATGAAAC	TAAAACGCAGCTCAGTAACAGTCCG	ann. 60 °C
			melt. 84 °C
IL-6	TTCACAAGTCCGGAGAGGAG	AGGAGAGCATTGGAAATTGG	ann. 60 °C
			melt. 78 °C
IL-8	CTGCTGGCTGTCCTTAACCT	ATTGGGCCAACAGTAGCCTT	ann. 60 °C
			melt. 78 °C
p16^INK4a^	AGGGCCGTGTGCATGACGTG	CATCGCGCACATCCAGCCGA	ann. 60 °C
			melt. 86 °C
p21^CIP1^	GCTCATGGCGGGCTGTCTCC	CCGGGGAATCTTCAGGCGC	ann. 60 °C
			melt. 86 °C

^1^ ann.: annealing; melt.: melting.

## Data Availability

Data are available upon reasonable request from the corresponding author.

## Abbreviations

The following abbreviations are used in this manuscript:

CAP	Cold atmospheric plasma
PDT	Photodynamic therapy
ALA	5-aminolaevulinic acid
AK	Actinic keratosis
SCC	Squamous cell carcinoma
UV-B	Ultraviolet B
ROS	Reactive oxygen species
RONS	Reactive oxygen and nitrogen species
SMD	Surface micro-discharge
IL-6	Interleukin-6
IL-8	Interleukin-8
AEC	3-Amino-9-Ethylcarbazole
TUNEL	Terminal deoxynucleotidyl transferase (TdT) dUTP Nick-End Labeling
PpIX	Protoporphyrin IX
